# Reduction of IL-17A Might Suppress the Th1 Response and Promote the Th2 Response by Boosting the Function of Treg Cells during Silica-Induced Inflammatory Response *In Vitro*

**DOI:** 10.1155/2014/570894

**Published:** 2014-02-16

**Authors:** Wen Tang, Fangwei Liu, Ying Chen, Laiyu Song, Wujing Dai, Chao Li, Dong Weng, Jie Chen

**Affiliations:** Division of Pneumoconiosis, School of Public Health, China Medical University, No. 92 North 2nd Road, Heping District, Shenyang, Liaoning 110001, China

## Abstract

Silica inhalation can induce chronic lung inflammation and fibrosis. Upon silica stimulation, activated macrophages trigger the T-lymphocyte which can differentiate into many different types of Th cells, including the recently discovered Th17 cells. IL-17A, the typical Th17 cytokine, is reported in some inflammatory diseases. However, the role of IL-17A in silica-induced inflammatory response is still not clear. The regulatory mechanism of silica-induced Th17 response also needs to be investigated. So we established a mice primary cell coculture system (macrophage and lymphocyte) to investigate the role of IL-17A in silica-induced inflammatory response *in vitro*, by using anti-IL-17A mAb and IL-1Ra. Both anti-IL-17A mAb and IL-1Ra decreased the level of IL-17A and increased the function of Treg cells. The Th1 response was suppressed and the Th2 response was promoted by the addition of anti-IL-17A mAb or IL-1Ra. IL-1Ra treatment decreased the level of IL-6, whereas the levels of IL-23 and ROR-**γ**t were increased. Our study demonstrated that IL-17A reduction altered the pattern of silica-induced Th responses by boosting the function of Treg cells *in vitro*. Blocking the function of IL-1 signal pathway could suppress the level of IL-17A, which played the major role in modulating silica-induced Th responses *in vitro*.

## 1. Introduction

Silica is commonly found in nature as sand or quartz. The inhalation of silica can induce chronic lung inflammation and fibrosis [1]. Upon stimulation by silica, alveolar macrophages are activated through the NLRP3 inflammasome, leading to some proinflammatory cytokines being modified into their mature forms, such as IL-1*β* and TNF-*α* [2, 3]. Then, the activated macrophages can regulate the T-lymphocyte response and promote the secretions of lymphocyte-derived cytokines (IL-4, IL-13, and IFN-*γ*) by performing their antigen presenting activity *in vitro* [4, 5].

Animal studies have shown that CD4+ T cells are the major T helper (Th) cells involved in the silica-induced lung inflammatory response [6]. CD4+ T cells can differentiate into several types, such as Th1, Th2, and regulatory T cells (Treg cells), characterized by their typical transcription factors T-bet, GATA3, and Foxp3, respectively [7]. The Th1 immune response is firstly enhanced after silica inhalation in the early inflammation stage. The secretions and expressions of Th1 cytokines, such as IL-2 and IFN-*γ*, are elevated [8, 9]. Then, with the development of inflammation and fibrosis, Th2 immune response is gradually enhanced to be predominant. The secretions and expressions of Th2 cytokines get increased, such as IL-4 and IL-13 [10, 11]. There is a consecutive balance between Th1 immune response and Th2 immune response [12]. And this Th1/Th2 balance is considered to be adjusted by Treg cells, which can attenuate the activity of effector T cells (Teff) by secreting IL-10 and TGF-*β* [13, 14].

The recently discovered Th17 cells are reported to mediate early lung inflammation in experimental silicosis [15]. IL-17, especially IL-17A, is known as the major Th17 cytokine. And ROR-*γ*t is the typical transcription factor for Th17 cells. IL-6, together with TGF-*β*, has been identified to induce the differentiation of Th17 cells and the expression of ROR-*γ*t [16, 17]. The proinflammatory cytokine IL-1*β* also plays a critical role in the early stage of Th17 cells differentiation [18]. Besides, Th17 cells are reported to express a higher level of IL-1 type I receptor (IL-1RI) than other T cell subsets [19]. So IL-1*β* takes part in the expansion of Th17 cells, especially in synergy with IL-23 [20]. Meanwhile, IL-1*β* regulates ROR-*γ*t expression during Th17 cells polarization and maintains cytokine productions of effector Th17 cells, such as IL-17A [21]. IL-17A is the typical proinflammatory cytokine produced by Th17 cells and can induce many inflammatory cytokines. Thus, IL-17A plays an important role in triggering inflammatory responses [22, 23]. There is a reciprocal developmental pathway for Treg cells and Th17 cells [16, 24]. The function of IL-17A can be abolished by neutralization with anti-IL-17A mAb [25]. The blockage of IL-17 causes a rapid increase in the number of Treg cells in the inguinal and popliteal lymph nodes of mice [26]. Blocking some key factors in the differentiation of Th17 cells can also suppress IL-17 function. Studies show that a functional IL-1RI is required for Th17 response [19]. The differentiation of Th17 cells is upregulated by IL-1*β* [27, 28]. IL-1Ra (Anakinra) can block the IL-1*β*/IL-1RI pathway by binding to IL-1R competitively, without generating any signal [29]. Therefore, anti-IL-17A mAb and IL-1Ra are used to influence IL-17A on either the secretion level or the expression level.

The objective of our study was to investigate the role of IL-17A in the silica-induced inflammatory response *in vitro*. In this study, we established a macrophage and lymphocyte coculture system *in vitro*, and silica was used to induce inflammatory response. Anti-IL17A mAb and IL-1Ra were added into the coculture system to influence the level of IL-17A. Our study demonstrated that IL-17A and Th17 cells were involved in silica-induced immune response *in vitro*. The reduction of IL-17A could modulate the Th1/Th2 responses by boosting the function of Treg cells. Blocking the function of IL-1 signal pathway could suppress the level of IL-17A, which played the major role in modulating silica-induced Th responses *in vitro*.

## 2. Materials and Methods

### 2.1. Animal

Healthy female C57BL/6 mice at 6–8 weeks of age were purchased from the Center of Experimental Animals, China Medical University (Shenyang, China). All animals were housed in a specific-pathogen-free environment and maintained on standard mouse chow with free access to food and water. All animal experiments were approved by the Animal Care and Use Committee at the China Medical University with a permit number of CMU62043013, which complies with the National Institute of Health Guide for the Care and Use of Laboratory Animals.

### 2.2. Cell Preparation

#### 2.2.1. Macrophage Isolation

After mice were sacrificed, the lungs were removed and washed twice with cold PBS. Bronchoalveolar lavage (BALF) was conducted several times by cannulating the trachea, injecting, and retrieving 1 mL aliquots of sterile physiological saline to get 6 mL BALF in total. The BALF was centrifuged at 800 g for 8 min at 4°C. The pellet was washed and resuspended in 1 mL RPMI 1640 (Thermo Fisher Scientific, Waltham, MA, USA). The total cell counts were determined using Cell Counting Chamber according to standard hematologic procedures. Cells were resuspended in RPMI 1640 containing 10% heat-inactivated fetal bovine serum (FBS Biological Industries, Kibbutz Beit-Haemek, Israel) and incubated for 2 h in 12-well plate. All the macrophages adhered to the plates after incubation.

#### 2.2.2. Lymphocyte Isolation

After being removed from the sacrificed mice, spleens were ground and disrupted mechanically in 5 mL 1X Mouse Lymphocyte Separation Medium (DKW33-R0100, EZ-SepTM, Dakewe, Shenzhen, China) in 35 mm dishes. Then the total dissociated splenocytes and the separation medium were transferred to 15 mL conical tubes. 200–250 *μ*L RPMI-1640 were added on top of the liquid. After being centrifuged for 30 min at 800 g, the lymphocyte layers were transferred into other 15 mL conical tubes. The isolated lymphocytes were washed with 10 mL RPMI-1640 and resuspended in PRMI-1640 containing 10%FBS. The total cell counts were determined using Cell Counting Chamber according to standard hematologic procedures.

### 2.3. Cell Coculture and Treatment

Crystalline silica particles were obtained from U.S. Silica Company (Min-U-Sil 5, Berkeley Springs, WV, USA). The silica particle size was 97% <5 *μ*m diameter, 80% <3 *μ*m diameter and a median diameter of 1.4 *μ*m. The nature of silica particles has already been tested by many studies [30, 31]. 5 mg/mL silica suspension was freshly made before use.

#### 2.3.1. Pretreatment on Macrophages

All the macrophages were divided into 4 groups: PBS group, silica group, silica + IL-1Ra group, and silica + anti-IL-17A group. Each group had one tube of 2 mL macrophages suspension. The macrophages from silica + IL-1Ra group and silica + anti-IL-17A group were rotated with 15 *μ*g/mL IL-1Ra (Biovitrum, Stockholm, Sweden) or 20 *μ*g/mL anti-IL-17A mAb (clone eBioMM17F3, eBioscience, San Diego, CA, USA), respectively, for 15 min. The macrophages from PBS group and silica group were rotated with PBS at equal volume. Then 16 *μ*L PBS (the same volume of silica suspension) was added to PBS group and 40 *μ*g/mL silica suspension was added to the other 3 groups each. The macrophages of 4 groups continued rotating for 45 min, and then the macrophages were placed into 12-well plates. All the macrophages were incubated for 2 h in 37°C with 5% CO_2_ to adhere to the plates.

#### 2.3.2. Pretreatment on Lymphocytes

The lymphocytes were also divided into 4 groups: PBS group, silica group, silica + IL-1Ra group, and silica + anti-IL-17A group. Each group had one tube of 2 mL lymphocytes suspension. The lymphocytes from silica + IL-1Ra group, and silica + anti-IL-17A group were treated with 30 *μ*g/mL IL-1Ra or 40 *μ*g/mL anti-IL-17A mAb, respectively, for 30 min.

#### 2.3.3. Coculture of Macrophages and Lymphocytes

The supernatant of pretreated macrophages was removed after being cultured for 2 h, and then the pretreated lymphocytes of each group were added to the wells of macrophages with the same group name, respectively. 2 × 10^5^ macrophages and 1 × 10^7^ lymphocytes were cocultured for 24 h or 48 h under the following condition: 40 *μ*g silica suspension for silica group, 40 *μ*g/mL silica suspension + 15 *μ*g/mL IL-1Ra for silica + IL-1Ra group, and 40 *μ*g/mL silica suspension + 20 *μ*g/mL anti-IL-17A mAb for silica + anti-IL-17A group. The PBS group was cultured with PBS at equal volume. The liquid sample was collected at two time points and then centrifuged at 800 g for 8 min at 4°C. The cell pellet, which mainly refers to lymphocytes, was used for mRNA extraction. The supernatant was stored at −70°C for ELISA test. These courses were repeated five times to confirm the findings.

### 2.4. mRNA Extraction and Real-Time RT-PCR

The total mRNA of cells was extracted using the Trizol reagent (Invitrogen, Carlsbad, CA, USA) according to the manufacturer's protocol. The mRNA concentration and the ratio of A 260/280 were determined by UV spectrophotometer. 2 *μ*g total mRNA was reverse transcribed in a volume of 20 *μ*L following the program: 37°C for 15 min and 85°C for 5 s. 2 *μ*L of cDNA was used in a 25 *μ*L PCR volume. Each sample was assayed in triplicate. The difference of the amplification efficiency between the target gene and the housekeeping gene was identified by comparing the slopes of the standard curves.

SYBR Green and Taqman methods were used for the real-time PCR assays. The PCR reactions were run on ABI 7500 (Applied Biosystems, Carlsbad, California, USA) using the two following programs: 95°C for 30 s, and 40 cycles of 95°C for 5 s, and 60°C for 34 s, or 95°C for 30 s, and 40 cycles of 95°C for 5 s, and 62°C for 34 s. Analysis was performed using the ABI 7500 system software (Applied Biosystems, Carlsbad, CA, USA). The primers and the probes were designed with the Primer3 (http://frodo.wi.mit.edu/primer3/), and the sequences were blasted (http://blast.ncbi.nlm.nih.gov/Blast.cgi). PrimeScript RT-PCR kit (DRR061A, Takara, Japan) was used for real-time RT-PCR. The primer sequences were as shown in Table 1. The probe sequences were as shown in Table 2.

### 2.5. ELISA Assay of Cytokines in Supernatant

The ELISA plate was first coated with 100 *μ*L capture antibody in coating buffer per well of ELISA kit (eBioscience, San Diego, CA, USA) and incubated overnight at 4°C. After being washed with 250 *μ*L wash buffer, each well was blocked and incubated for 1 h at room temperature (RT). Then 100 *μ*L supernatant or the different dilutions of standard (for standard curve) were added to each well, incubated for 2 h at RT. The well was incubated with 100 *μ*L detection antibody for 1 h at RT, followed by incubation with 100 *μ*L avidin-HRP for 30 min at RT. Finally 100 *μ*L substrate solution was added to each well to incubate for 15 min at RT. 50 *μ*L stop solution was used to stop the reaction. The plate was read at 450 nm and analyzed. The ELISA was performed in triplicate.

### 2.6. Statistical Analyses

The SPSS 17.0 software was used to conduct statistical analyses. The differences between values were evaluated through a one-way analysis of variance (ANOVA) followed by pairwise comparison with the Student-Newman-Keuls test. *P* < 0.05 was considered statistically significant, and all values are means ± SEM.

## 3. Results

### 3.1. Anti-IL-17A mAb and IL-1Ra Decreased the Level of IL-17A after Silica Stimulation *In Vitro*

**Figure 1:**
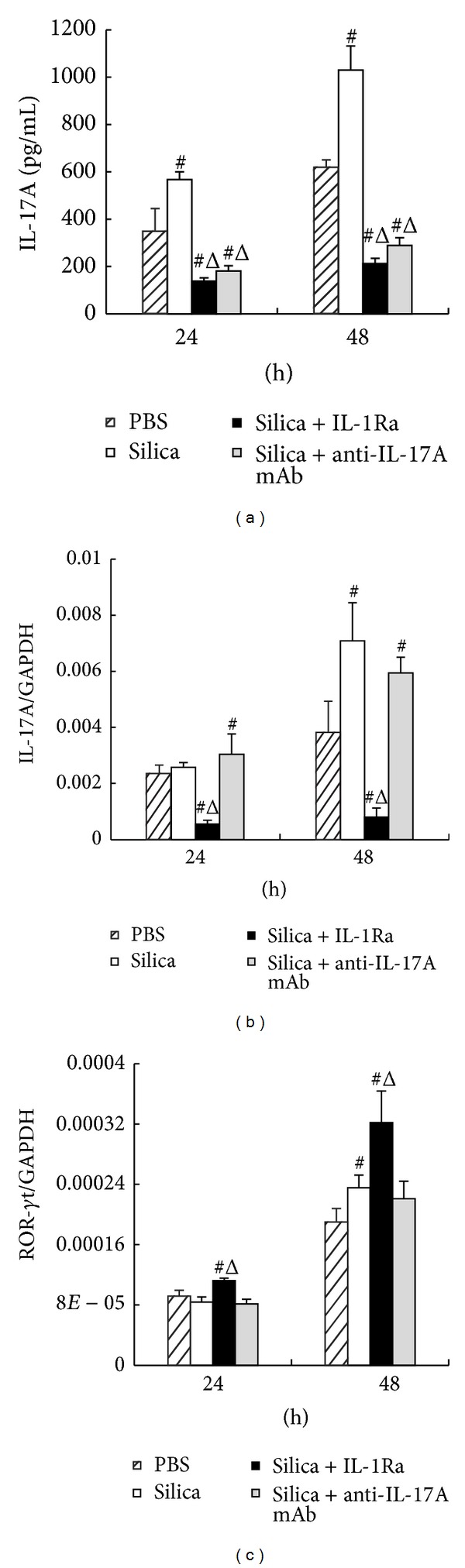
Anti-IL-17A mAb and IL-1Ra decreased the secretion of IL-17A in silica-induced Th response *in vitro*. (a) The secretion of IL-17A in supernatant of the macrophage-lymphocyte cocultured system was assayed by ELISA. ((b) and (c)) The expressions of IL-17A and ROR-*γ*t in lymphocytes were assayed by realtime PCR. The concentration of IL-1Ra is 15 *μ*g/mL and the concentration of anti-IL-17A mAb is 20 *μ*g/mL. Data were presented as mean ± SEM (*n* = 5). #: *P* < 0.05, significantly different compared with the PBS group. Δ: *P* < 0.05, significantly different compared with the silica group.

To testify whether IL-17A and Th17 response play a role in response to silica stimulation *in vitro*, anti-IL-17A mAb and IL-1Ra were added to the coculture system. The levels of IL-17A and ROR-*γ*t were examined using ELISA and real-time PCR. Silica stimulation increased the secretion of IL-17A at both time points compared with the PBS group (Figure 1(a)). The secretion of IL-17A was significantly lower in both the silica + anti-IL-17A mAb group and the silica + IL-1Ra group compared with the silica group (Figure 1(a)). Besides, the expression of IL-17A increased significantly in silica group at 48 h compared with the PBS group (Figure 1(b)). The IL-1Ra treatment significantly decreased the expression of IL-17A compared with either the PBS group or the silica group (Figure 1(b)). However, the addition of anti-IL-17A mAb did not decrease the expression of IL-17A compared with the silica group (Figure 1(b)). To further test Th17 response, we examined the expression of its typical transcription factor ROR-*γ*t. The silica stimulation increased the expression of ROR-*γ*t significantly at 48 h compared with the PBS group (Figure 1(c)). The anti-IL-17A mAb did not influence the expression of ROR-*γ*t (Figure 1(c)), whereas the expression of ROR-*γ*t increased significantly after IL-1Ra treatment compared with the silica group (Figure 1(c)).

### 3.2. Anti-IL-17A mAb and IL-1Ra Suppressed the Th1 Response and Promoted the Th2 Response

**Figure 2:**
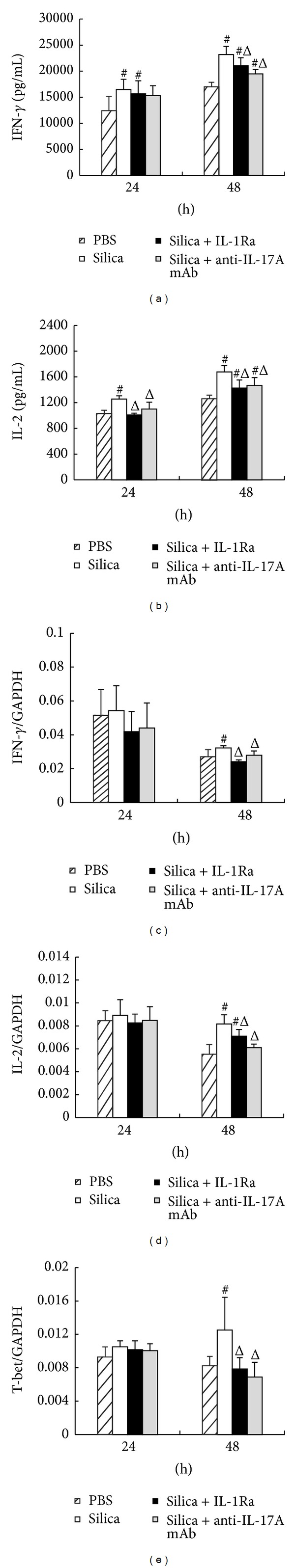
The Th1 response was suppressed by anti-IL-17A mAb and IL-1Ra treatments. ((a) and (b)) The secretions of IFN-*γ* and IL-2 in supernatant of the macrophage-lymphocyte cocultured system were assayed by ELISA. ((c), (d), and (e)) The expressions of IFN-*γ*, IL-2, and T-bet in lymphocytes were assayed by real-time PCR. The concentration of IL-1Ra is 15 *μ*g/mL and the concentration of anti-IL-17A mAb is 20 *μ*g/mL. Data were presented as mean ± SEM (*n* = 5). #: *P* < 0.05, significantly different compared with the PBS group. Δ: *P* < 0.05, significantly different compared with the silica group.

To study the effect of IL-17A and/or Th17 cells on Th1/Th2 response, we examined the secretions of Th1 (IFN-*γ*, IL-2) and Th2 (IL-4) cytokines in the supernatant of the cocultured system. Silica stimulation increased the levels of Th1 cytokines and its typical transcription factor T-bet (Figure 2). However, the secretion of IFN-*γ* significantly decreased in the silica + anti-IL-17A mAb group at 48 h compared with the silica group (Figure 2(a)). Besides, the secretion of IL-2 also decreased in the silica + anti-IL-17A mAb group at both time points compared with the silica group (Figure 2(b)). Real-time PCR assay confirmed the ELISA results of Th1 cytokines. The addition of anti-IL-17A mAb suppressed the expressions of IFN-*γ* and IL-2 at 48 h (Figures 2(c) and 2(d)). The expression of Th1 typical transcription factor T-bet was also examined by real-time-PCR. Anti-IL-17A mAb restricted the increase of the T-bet expression after silica stimulation (Figure 2(e)). The IL-1Ra imitated the effect of anti-IL-17A mAb by decreasing the secretions and expressions of Th1 cytokines and its transcription factor (Figure 2).

**Figure 3:**
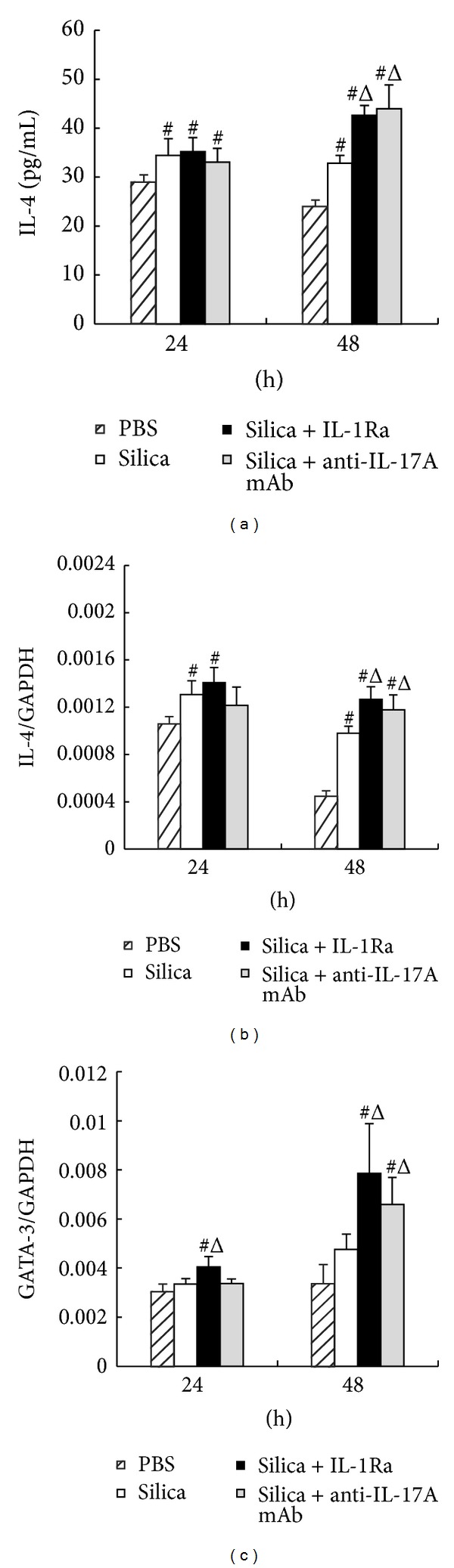
The Th2 response was promoted by anti-IL-17A mAb and IL-1Ra treatments. (a) The secretion of IL-4 in supernatant of the macrophage-lymphocyte cocultured system was assayed by ELISA. ((b) and (c)) The expressions of IL-4 and GATA-3 in lymphocytes were assayed by real-time PCR. The concentration of IL-1Ra is 15 *μ*g/mL and the concentration of anti-IL-17A mAb is 20 *μ*g/mL. Data were presented as mean ± SEM (*n* = 5). #: *P* < 0.05, significantly different compared with the PBS group. Δ: *P* < 0.05, significantly different compared with the silica group.

We also checked the Th2 cytokines and its typical transcription factor GATA-3 using the ELISA and real-time PCR assays. Silica stimulation increased the level of Th2 cytokine IL-4 significantly. The secretion and expression of IL-4 increased markedly in the silica + anti-IL-17A mAb group compared with the silica group at 48 h (Figures 3(a) and 3(b)). The expression of GATA-3 gained a slight increase in silica + anti-IL-17A mAb group compared with the silica group at 48 h (Figure 3(c)). The results of Th2 related factors in silica + IL-1Ra group were similar to those in silica+anti-IL-17A mAb group. IL-1Ra not only increased the secretion and expression of IL-4 significantly, but also stimulated the increase of GATA-3 expression even at both time points compared with silica + anti-IL-17A mAb group (Figure 3).

### 3.3. Anti-IL-17A mAb and IL-1Ra Might Boost the Function of Treg Cells

**Figure 4:**
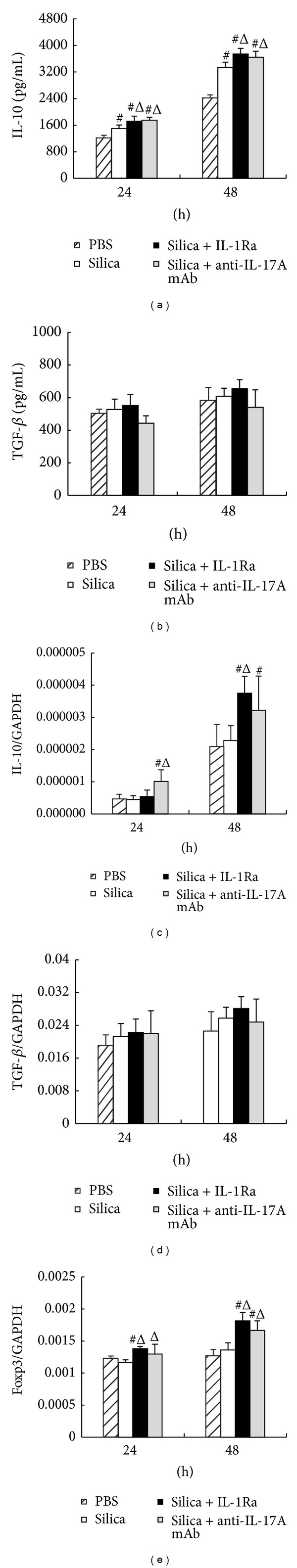
The function of Treg cells might be boosted by anti-IL-17A mAb and IL-1Ra. ((a) and (b)) The secretion of IL-10 and TGF-*β* in supernatant of the macrophage-lymphocyte cocultured system was assayed by ELISA. ((c), (d), and (e)) The expressions of IL-10, TGF-*β*, and Foxp3 in lymphocytes were assayed by real-time PCR. The concentration of IL-1Ra is 15 *μ*g/mL and the concentration of anti-IL-17A mAb is 20 *μ*g/mL. Data were presented as mean ± SEM (*n* = 5). #: *P*<0.05, significantly different compared with the PBS group. Δ: *P*<0.05, significantly different compared with the silica group.

To investigate the mechanism of how IL-17A influenced the Th1/Th2 immune response, we checked the Treg cells related factors, the transcription factor Foxp3, and negative regulatory cytokines IL-10 and TGF-*β*. The secretion and expression of IL-10 in silica + anti-IL-17A mAb group increased significantly at 24 h compared with the silica group (Figures 4(a) and 4(c)), whereas there was no significant difference in the secretion and expression of TGF-*β* between these two groups (Figures 4(b) and 4(d)). In addition, the expression of Foxp3 increased significantly in silica + anti-IL-17A mAb group compared with the silica group at both time points (Figure 4(e)). The IL-1Ra treatment mimicked the effect of anti-IL-17A. The secretion and expression of IL-10 in silica + IL-1Ra group also increased significantly at 48 h compared with the silica group (Figures 4(a) and 4(c)). The expression of Foxp3 increased significantly in silica + IL-1Ra group compared with the silica group at 24 h and 48 h (Figure 4(e)).

### 3.4. IL-17A Modulated the Silica-Induced Th Responses *In Vitro*

**Figure 5:**
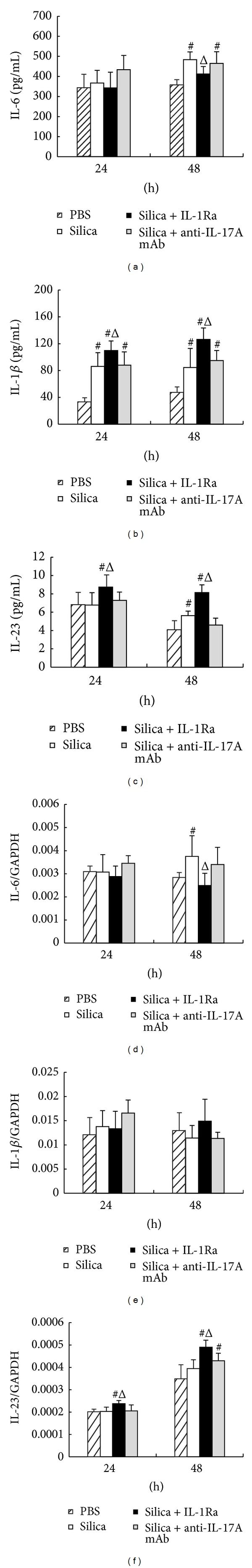
Th17 differentiation-related cytokines were regulated by IL-1Ra treatment. ((a), (b), and (c)) The secretions of IL-6, IL-1*β*, and IL-23 in supernatant of the macrophage-lymphocyte cocultured system were assayed by ELISA. ((d), (e), and (f)) The expressions of IL-6, IL-1*β*, and IL-23 in lymphocytes were assayed by real-time PCR. The concentration of IL-1Ra is 15 *μ*g/mL and the concentration of anti-IL-17A mAb is 20 *μ*g/mL. Data were presented as mean ± SEM (*n* = 5). #: *P* < 0.05, significantly different compared with the PBS group. Δ: *P* < 0.05, significantly different compared with the silica group.

To further study whether IL-17A or Th17 cells regulated the Th1/Th2 response by boosting the Treg cells function, we examined some cytokines that were important for Th17 cells differentiation and proliferation. IL-6 was considered to promote the naïve T cells to differentiate into Th17 cells pathways [32]. IL-1*β* and IL-23 further promoted and stabilized the generation of Th17 cells [20, 33]. According to the ELISA and real-time PCR assays, the secretion and the expression of IL-6 decreased significantly in silica + IL-1Ra group compared with the silica group at 48 h (Figure 5(a)), which meant that IL-1Ra inhibited the expression of IL-6. In contrast, the secretion of IL-1*β* apparently increased after being treated with IL-1Ra compared with simple silica induction (Figure 5(b)). Similarly, the secretion and expression of IL-23 increased significantly in silica + IL-1Ra group compared with the silica group at 24 h and 48 h (Figures 5(c) and 5(f)). There was no significant difference in the expression of IL-1*β* between these two groups (Figure 5(e)). Although IL-1Ra suppressed the level of IL-6, the level of IL-23 got increased after IL-1Ra treatment.

## 4. Discussion

**Figure 6:**
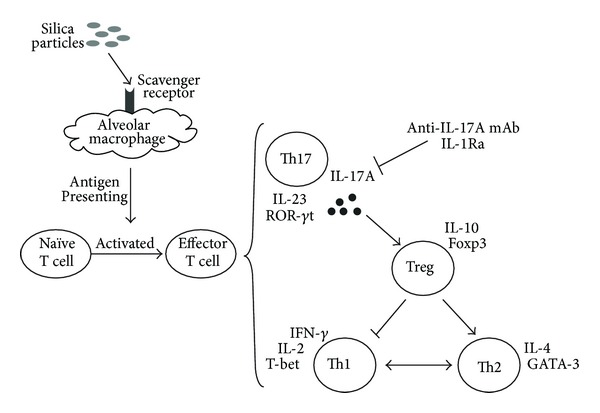
A schematic representation for the silica-induced IL-17A release and the changes of Th immune responses caused by IL-1Ra and anti-IL-17A mAb.

Silica inhalation results in chronic lung inflammation and fibrosis according to many studies. When silica particles invade our immune system, alveolar macrophages can bind to the silica particles through the MARCO scavenger receptor as the first line of defense [34]. First of all, macrophages can recruit many inflammatory cells, such as neutrophils and lymphocytes. Meanwhile, macrophages can also release some proinflammatory cytokines upon silica stimulation, such as IL-1*β* and TNF-*α* [2, 3]. In addition, macrophages also play an important role in antigens presenting, which may induce the activation of naïve T cells and different Th immune responses [4]. Our previous animal study showed that IL-17A might promote the silica-induced early lung inflammation. To further study the role of IL-17A during silica-induced inflammatory response *in vitro*, we established the coculture system of macrophages and lymphocytes. The aim of this study was to explore the role of IL-17A during silica-induced immune response *in vitro*. Our results showed that silica stimulation increased the level of IL-17A and ROR-*γ*t, which indicated that IL-17A and Th17 response took part in silica-induced immune response *in vitro*. The addition of anti-IL-17A mAb and IL-1Ra reduced the level of IL-17A significantly. Anti-IL-17A mAb neutralized the secreted IL-17A. IL-1Ra restricted not only the secretion of IL-17A, but also the expression of IL-17A. Both anti-IL-17A mAb and IL-1Ra might suppress the Th1 response and enhance the Th2 response by boosting the function of Treg cells. IL-1Ra just inhibited the level of IL-17A. But the Th17 related factors, such as ROR-*γ*t and IL-23, were not suppressed by the IL-1Ra (Figure 6).

In this study, in order to discuss the role of IL-17A in silica-induced immune response anti-IL-17A mAb was used to neutralize the IL-17A *in vitro*. After silica stimulation, the expression and secretion of IL-17A were enhanced as well as the expression of ROR-*γ*t, which was consistent with the study *in vivo* showing that Th17 response increased in the silica-induced lung fibrosis [15]. Meanwhile, IL-1*β*, IL-6 and IL-23 contributed to the enhancement of Th17 response after silica stimulation [17, 22, 35]. Besides, the traditional Th1 and Th2 immune responses enhanced by silica stimulation and Treg cells also got promoted. The increase of Th17 related factors indicated that silica could induce Th17 response, as well as the Th1/Th2 response. And IL-17A took part in silica-induced Th response.

It was shown that Treg cells and Th17 cells shared a reciprocal developmental pathway [16, 24]. In this experiment, the secretion of IL-17A reduced significantly by the addition of anti-IL-17A mAb. The secretion of IL-10 and the expression of Foxp3 were promoted when IL-17A decreased. IL-10 was the major inhibitory cytokine by which Treg cells regulated the immune system. Foxp3 was the characterized nuclear transcription factor of Treg cells [14, 36]. IL-17A reduction actually enhanced the function of Treg cells to some extent. Treg cells played a critical role in maintaining self-tolerance as well as in regulating immune responses, mainly secreting two anti-inflammatory cytokines: IL-10 and TGF-*β*; however, the TGF-*β* was not absolutely required for suppression *in vitro* [7, 37]. Besides, there was a key balance between Th1 and Th2 type responses [12]. And our previous study showed that Treg cells could regulate the balance of Th1/Th2 immune response through suppressing the Th1 response in silica-induced lung fibrosis *in vivo* [13]. In this experiment, IL-17A reduction decreased Th1 type cytokines (IFN-*γ*, IL-2) and its transcription factor T-bet, whereas increased the Th2 type cytokine (IL-4) and its transcription factor GATA-3. The change of the Th1/Th2 response model *in vitro* might also be regulated by Treg cells.

IL-1Ra could negatively regulate function of IL-1*β* by competitively binding to IL-1RI without IL-1*β* signal generation [21]. IL-1*β* signaling played an important role in the secretion of IL-17A [33, 38, 39]. So IL-1Ra was used to influence the level of IL-17A. Our results showed that the expression and secretion of IL-17A decreased significantly after IL-1Ra treatment. And IL-1Ra made an imitated effect by restricting the expression of IL-17A and influencing Treg cells. The expression of IL-10 and Foxp3 got promoted after IL-1Ra treatment, indicating that the function of Treg cells was enhanced. And the increased Treg cells could regulate Th immune response by suppressing Th1 response and promoting Th2 response. Besides, the absence of IL-1 signaling could also reduce the secretion of Th1 cytokines. The production of typical Th1 cytokine decreased in the supernatants of cultured lymphocytes derived from IL-1RI -/- and IL-1a/b-/- mice after immunization with mBSA [28]. The addition of IL-1Ra in our cocultured system suppressed the secretion and expression of Th1 cytokines and its typical transcription factor T-bet. Meanwhile, decreased IL-2, IFN-*γ*, and T-bet could improve the Th2 differentiation and IL-4 production [40, 41], so the Th2 cytokines and its typical transcription factor GATA-3 were elevated after IL-1Ra treatment. Therefore, the change of Th1/Th2 pattern that followed the IL-1Ra treatment could be attributed to two aspects, the enhanced function of Treg cells and/or the absence of IL-1 signaling.

Although both the anti-IL-17A mAb and IL-1Ra influenced the secretion of IL-17A *in vitro*, they made the different impacts on Th17 response. The anti-IL-17A mAb simply neutralized secreted IL-17A, without changing the expression of IL-17A, even the Th17 differentiation-related factors. However, the IL-1Ra treatment not only reduced both the secretion and the expression of IL-17A by blocking the function of IL-1*β*, but also affected the Th17 differentiation-related factors, such as IL-6, IL-23, and ROR-*γ*t. On the one hand, the proinflammatory cytokines IL-1*β* or IL-6 could trigger IL-17A cytokine production *in vitro* [42–44]. The silica-induced IL-6 expression of pneumocytes was mainly mediated via IL-1*β*. And the level of IL-6 could reduce effectively after being treated with IL-1Ra *in vitro* [45], which was consistent with our results. So IL-1Ra could reduce the level of IL-17A by restricting the expression of IL-6. On the other hand, the level of ROR-*γ*t, the key transcription factor of Th17 cells, increased significantly after IL-1Ra treatment in our coculture system. The animal study also confirmed that ROR-*γ*t increased in the silica-induced lung fibrosis *in vivo* [15]. The increased ROR-*γ*t expression could be the result of the increased level of IL-23, which contributed to the expression of ROR-*γ*t and the Th17 cells differentiation [35, 42, 46]. Furthermore, it was reported that there was a balance between Th17 and Th1 and the decreased Th1 response in our cocultured system also may result in the increase of ROR-*γ*t. Consequently, although the IL-1Ra suppressed the levels of IL-17A and IL-6, the expressions of ROR-*γ*t and IL-23 still increased. This suggested that IL-1 signal was important to the Th17 cells differentiation and IL-17A secretion, but its absence might be compensated by the increased level of IL-23 in the expression of ROR-*γ*t.

## 5. Conclusions

Our study demonstrated that IL-17A and Th17 cells were involved in silica-induced immune response *in vitro*. IL-17A reduction could suppress the Th1 response and promote the Th2 response by boosting the function of Treg cells. Blocking the function of IL-1 signal pathway could suppress the level of IL-17A, which played a critical role in modulating the silica-induced Th responses *in vitro*.

## Figures and Tables

**Table 1:** 

T-bet	
Sense	5′-TCAACCAGCACCAGACAGAGA-3′,
Antisense	5′-TCCACCAAGACCACATCCAC-3′.
GATA-3	
Sense	5′-GAAACCGGAAGATGTCTAGCAAA-3′,
Antisense	5′-TGGAGTGGCTGAAGGGAGA-3′.
Foxp3	
Sense	5′-CAGCTCTGCTGGCGAAAGTG-3′,
Antisense	5′-TCGTCTGAAGGCAGAGTCAGGA-3′.
ROR-*γ*t	
Sense	5′-ACGGCCCTGGTTCTCATCA-3′,
Antisense	5′-CCAAATTGTATTGCAGATGTTCCAC-3′.
IL-23	
Sense	5′-ACATGCACCAG CGGGACATA-3′,
Antisense	5′-CTTTGAAGATGTCAGAGTCAAGCAG-3′.
IL-2	
Sense	5′-TTGAGTGCCAATTCGATGATGAG-3′,
Antisense	5′-TTGAGATGATGCTTTGACAGAAGG-3′.
IFN-*γ*	
Sense	5′-AAGCGTCATTGAATCACACCTG-3′,
Antisense	5′-TGACCTCAAACTTGGCAATACTC-3′.
IL-4	
Sense	5′-AAAATCACTTGAGAGAGATCATCGG-3′,
Antisense	5′-GTTGCTGTGAGGACGTTTGG-3′.
IL-10	
Sense	5′-GGGGCCAGTACAGC CGGGAA-3′,
Antisense	5′-CTGGCTGAAGGCAGTCCGCA-3′.
TGF-*β*1	
Sense	5′-TGTGGAACTCTACCAGAAATATAGC-3′,
Antisense	5′-GAAAGCCCTGTATTCCGTCTC-3′.
IL-17	
Sense	5′-GCAAAAGTGAGCTCCAGAAGG-3′
Antisense	5′-TCTTCATTGCGGTGGAGAGTC-3′.
IL-1*β*	
Sense	5′-TGACCTGGGCTGTCCTGATG-3′,
Antisense	5′-GGTGCTCATGTCCTCATCCTG-3′.
IL-6	
Sense	5′-CAATTCCAGAAACCGCATGAAG-3′,
Antisense	5′-GTAGGGAAGGCCGTGGTTG-3′.
GAPDH	
Sense	5′-CAATGTGTCCGTCGTGGATCT-3′,
Antisense	5′-GTCCTCAGTGTAGCCCAAGATG-3′

**Table 2:** 

IL-2	5′-(FAM) CCTCAGAAAGTCCACCACAGTTGCT (BHQ1)-3′.
IFN-*γ*	5′-(FAM) CTTCTTCAGCAACAGCAAGGCGAA (BHQ1)-3′.
IL-4	5′-(FAM) TGGCGTCCCTTCTCCTGTGACCTCG (BHQ1)-3′.
IL-10	5′-(FAM) GCACCCACTTCCCAGTCGGCCAGAGCC (BHQ1)-3′.
TGF-*β*1	5′-(FAM) TTCAGCCACTGCCGTACAACTCCAG (BHQ1)-3′.
IL-17	5′-(FAM) CCTCAGACTACCTCAACCGTTCCAC (BHQ1)-3′.
IL-1*β*	5′-(FAM) TCGCAGCAGCACATCAACAAGAGC (BHQ1)-3′.
IL-6	5′-(FAM) CACCAGCATCAGTCCCAAGAAGGCA (BHQ1)-3′.
GAPDH	5′-(FAM) CGTGCCGCCTGGAGAAACCTGCC (BHQ1)-3′.

## Abbreviations

IL-17A: Interleukin-17A
IL-1Ra: Interleukin-1 receptor antagonist
IL-6: Interleukin-6
IL-23: Interleukin-23
IL-1RI: Interleukin-1 receptor, type I
IL-1: Interleukin-1
IL-1*β*: Interleukin-1 beta
IL-4: Interleukin-4
IL-13: Interleukin-13
IL-10: Interleukin-10
TNF-*α*: Tumor necrosis factor-alpha
NLRP3: NOD-like receptor family, pyrin domain containing 3
IFN-*γ*: Interferon-gamma
Treg: Regulatory T cells
Th1: T helper type 1 cells
Th2: T helper type 2 cells
Th17: T helper type 17 cells
Teff: Effector T cell
T-bet: T-cell-specific T-box transcription factor
GATA-3: GATA binding protein 3
Foxp3: Forkhead box P3
ROR-*γ*t: RAR-related orphan receptor-gamma
TGF-*β*: Transforming growth factor-beta
CD4: Cluster of Differentiation 4 receptors
MARCO: Macrophage receptor with collagenous structure.
